# Determination of Glyphosate and AMPA in Food Samples Using Membrane Extraction Technique for Analytes Preconcentration

**DOI:** 10.3390/membranes12010020

**Published:** 2021-12-24

**Authors:** Katarzyna Gębura, Piotr P. Wieczorek, Anna Poliwoda

**Affiliations:** 1 Łukasiewicz Research Network—Institute of Heavy Organic Synthesis “Blachownia”, Energetyków 9, 47-225 Kedzierzyn-Kozle, Poland; katarzyna.gebura@icso.lukasiewicz.gov.pl; 2 Faculty of Chemistry, University of Opole, Pl. Kopernika 11a, 45-040 Opole, Poland; Piotr.Wieczorek@uni.opole.pl

**Keywords:** glyphosate and AMPA, supported liquid membrane extraction, ultrasonic-assisted solvent extraction, food samples

## Abstract

The method for determining glyphosate (NPG) and its metabolite AMPA (aminomethyl phosphonic acid) in solid food samples using UAE-SLM-HPLC–PDA technique was developed. Firstly, ultrasonic-assisted solvent extraction (UAE) and protein precipitation step were used for the analyte isolation. Then, the supernatant was evaporated to dryness and redissolved in distilled water (100 mL). The obtained solution was alkalized to pH 11 (with 1 M NaOH) and used directly as donor phase in SLM (supported liquid membrane) extraction. The SLM extraction was performed using 2 M NaCl (5 mL) as an acceptor phase. The flow rate of both phases (donor and acceptor) was set at 0.2 mL/min. The membrane extraction took 24 h but did not require any additional workload. Finally, the SLM extracts were analyzed using the HPLC technique with photo-diode array detector (PDA) and an application of pre-column derivatization with *p*-toluenesulfonyl chloride. Glyphosate residues were determined in food samples of walnuts, soybeans, barley and lentil samples. The LOD values obtained for the studied food were 0.002 μg g^−1^ and 0.021 μg g^−1^ for NPG and AMPA, respectively. Recoveries values ranged from 32% to 69% for NPG, 29% to 56% for AMPA and depended on the type of sample matrix. In the case of buckwheat and rice flour samples, the content of NPG and AMPA was below the detection level of a used analytical method.

## 1. Introduction

The rapid and continuous development of industry causes more and more chemicals to be released into the environment, contributing to the global pollution of the world around us [1]. These compounds get into the air, soil, groundwater and food, undergoing various chemical changes. At the same time, the progress of science has resulted in the fact that we are currently obtaining more and more information on the biological activity of foreign substances (xenobiotics) and their metabolites [2]. It turns out that these compounds mostly threaten living organisms, thus negatively affecting human life and health. One of the ways to reduce the adverse effects of xenobiotics on the human body is to monitor the environment for the presence of these substances constantly. This type of monitoring may enable the introduction of appropriate regulations and legal standards that define maximum admissible concentration (MAC) of harmful compounds in individual elements of the environment, which at the same time will constitute a safe level for living organisms.

One of the known groups of xenobiotics are compounds containing a phosphorus atom in their structure, which due to their diversity, are used in many areas of life. They have been applied as chemical weapons (poisonous warfare agents), in medicine as drugs, in industry, as complexing agents, and in agriculture as plant protection agents.

One of the most commonly used pesticides, and also a compound from the phosphonate group, is glyphosate (N-phosphonomethylglycine, NPG), which is a herbicide with a diverse spectrum of activity [3]. Because of the high energy required for dissociation, the bond C-P is very stable compared with O–P, N–P, or H–P ones. The presence of the C-P bond results in the organophosphonates resistance to chemical hydrolysis and thermal decomposition and thus significantly affects the durability of phosphonates in the environment [4]. However, it is relatively easy to biodegrade [5]. The action of glyphosate involves the inhibition of the enzyme 5-enolpyruvylshikimim-3-phosphate synthase (EPSP) of the shikimate pathway, which leads to plant death. The main product of NPG biodegradation is AMPA (aminomethylphosphonic acid), a chemical compound that is more toxic and more persistent than glyphosate [6,7]. Both substances are characterized by high polarity, good solubility in water but poor solubility in organic solvents [8]. The pesticides produced by NPG are widely used throughout the world; therefore, it generates concern about its long-term effects, and thus the environmental monitoring is necessary. Field studies show the half-life of glyphosate in soil ranges between a few days to several months, or even a year, depending on soil composition. The authors say the research demonstrates that soil sorption and degradation of glyphosate vary significantly depending on the soil’s physical, chemical, and biological properties. AMPA has been classified as persistent in soils, with a typical half-life of 151 days, but varying from 76 to 240 days and depending on field conditions [9,10].

Furthermore, many literature reports describe that glyphosate and its co-formulants (used in pesticides) can be included in the group of compounds that cause the endocrine effect [8,10,11,12,13,14,15,16,17,18,19,20,21]. However, the regulations regarding these compounds are constantly changing. Scientists are trying to prove the endocrine effect of NPG and it is co-formulants. For this reason, it is crucial to develop methods for NPG and AMPA analysis in real samples.

Despite many literature studies on the determination of glyphosate and AMPA in various matrices, the determination of these substances is still a challenge for analytical chemists. In many cases, available analytical procedures described in the literature often require laborious sample preparation steps and highly selective detection techniques (e.g., MS), which are expensive and not always widely available [18,22,23,24]. Therefore, as part of this study, attempts were made to develop and optimize NPG and AMPA determination in solid food samples. The analyzed matrices included walnut, soybean, buckwheat, barley, lentil and rice flour. The developed analytical procedure consisted of (1) ultrasonic-assisted solvent extraction (UAE) used for isolation step, (2) protein precipitation with acetone, (3) supported liquid membrane extraction procedure for analyte preconcentration and (4) HPLC—PDA technique applied for quantification and qualification analysis.

## 2. Materials and Methods

### 2.1. Chemicals

Aliquat 336 (methyltrioctylammonium chloride) used as a carrier, di-*n*-hexyl ether (DHE) a membrane diluent, derivatization reagent- *p*-toluenesulfonyl chloride (Tos-Cl) and AMPA (aminomethyl phosphonic acid) were obtained from Sigma-Aldrich (Poznań, Poland). Glyphosate *(N*-(phosphonomethyl) glycine, NPG) was isolated in our laboratory from a commercially available formulation—*Roundup^®^* 360 SL (Monsanto, MO, USA). The 31P-NMR technique confirmed the purity of the obtained compound. Acetonitrile of HPLC gradient grade was purchased from Merck (Warsaw, Poland). Inorganic salts and acids (KH_2_PO_4_, K_2_HPO_4_, H_3_PO_4_, NaCl and HCl) were supplied by Avantor Performance Materials Poland (Gliwice, Poland). All chemicals were of analytical grade. Water was purified with a Milli-Q-RO4 system (Millipore, Bedford, MA, USA).

The NPG and AMPA stock solution was prepared in purified water at concentration 0.6 mg L^−1^ and stored in a plastic flask. Analyzed compounds were more stable in plastic flask due to adsorption on the glass. The stability of the stock solutions was tested once a week by the HPLC-PDA system. All solutions were stored at 4 °C.

### 2.2. Samples

The analyzed food samples (walnut, soybean, buckwheat, barley, lentil and rice flour) were purchased from the regional market in Opole (Poland). All samples were raw and ground up. The complete profile of studied materials, obtained from labels of manufacturers, is presented in Table 1.

### 2.3. Extraction Procedures

Before SLM procedure the step with UAE extraction and protein precipitation was applied. In this case, spiked or unspiked solid samples (4 g) was extracted with 30 mL of water for 15 min using UAE method (temperature 24 °C, frequency 42 kHz, power 130 W). Than, the extract was centrifuged (room temperature, 5 min at 8500 rpm). In the next step, 10 mL of acetone was added to the obtained supernatant (25 mL) to precipitate the proteins, and again the solution was centrifuged for 5 min at 9000 rpm. After this stage, the acetone was evaporated from the supernatant of the sample and the obtained solution was placed in a plastic flask, diluted to 100 mL with water, adjusted to pH 11 with concentrated NaOH and used as donor phase for membrane extraction.

The used membrane system was the same as previously described in the literature by Khrolenko et al. [24]. Briefly, the porous PTFE (poly(tetrafluoroethylene)) membrane (Fluoropore membrane filters, pore size 0.2 μm, Merck Millipore, Darmstadt, Germany) was impregnated with 20% Aliquat 336 in DHE for 15 min for each side. The viscosity of the used membrane phase solvents (carrier and diluent) has a prominent influence on the membrane transport properties, especially ions diffusivity and SLM stability. In SLM, a hydrophobic porous polymeric membrane holds the membrane liquid (membrane phase) into its micro-pores by capillary action. It has been proved that high viscosity (up to 500 cP) may slow down the displacement of the liquids from the micron pores under pressure and improve the SLM stability [25]. Aliquat 336 is used as a complex with counter ions (chlorides) to allow it to be dissolved in the hydrophobic organic phase (di-n-hexyl ether (DHE), a membrane diluent). The time of membrane impregnation was experimentally tested at our research group earlier, and the proposed of 15 min was enough. Moreover, to determine the amount of membrane phase immobilized in the supporting membrane, the membranes were weighted before and after impregnation to monitor the effectivity of the impregnation methodology. Then, the membrane was taken between two blocks made of PVDF (poly(vinylidene difluoride). Before extraction, the excess organic solvent was removed by rinsing the membrane with water through both channels. The applied donor phase volume was 100 mL, whereas the acceptor phase was 5 mL. The SLM extraction was performed using a syringe pump, a model Gilson Miniplus 3 (Gilson S.A., Villiers-le-Bel, France). In the first step, 100 mL of aqueous sample solution (spiked and unspiked, pH 11) were pumped through the donor channel at a flow rate of 0.2 mL min^−1^. At the same time, the acceptor phase consisted of 5 mL of 2 M NaCl, circulating in the acceptor channel with the same flow rate. After the extraction (which takes 24 h), the acceptor phase was taken for the derivatization procedure and transferred (off-line) into the HPLC system. Extraction efficiency was calculated from the following equation: E = (C_A_V_A_/C_D_V_D_)·100%, where: C_A_, and C_D_ are the studied analyte concentrations in donor and acceptor phases, respectively. V_A_ and V_D_ are the appropriate phase volumes. Whereas, recovery (R) was calculated according to the following formula R = n_A_/n_Di_ − n_Df_, where n_A_ are numbers of analyte moles in acceptor phase, n_Di_—initial numbers of analyte moles in donor phase, n_Df_—final numbers of analyte moles in donor phase.

### 2.4. HPLC Analysis

The procedure of derivatization was also used from the previous research [24]. However, it was slightly modified. When acid was used as the receiving phase, before the derivatization step, 10 mL of acceptor was neutralized (up to pH 7) with concentrated KOH solution. In our case, the acceptor phase was a NaCl solution instead of HCL, so this step was omitted. For the derivatization process: 1500 µL of acceptor phase was taken and mixed with 750 µL of 0.4 M phosphate buffer (pH 11) and 300 µL of *p-*toluenesulfonyl chloride solution (10 mg mL^−1^ in acetonitrile) and then heated for 10 min in a water bath at 50 °C. After that, 300 µL of 1 M HCL was added to neutralized solution, and the HPLC analysis was performed. The detection was made at 240 nm wavelength.

The HPLC system DionexUltiMate 3000 with PDA detector equipped with software Chromeleon v. 6.80 Dionex was used to separate the analyzed compounds. Gradient separations were performed on Microsorb-MV, C18 column (250 mm × 4.6 mm × 5 µm) from Sigma-Aldrich (Poznań, Poland). The mobile phase consisted of a mixture of 10 mM phosphate buffer (KH_2_PO_4_ adjusted to pH 2.3 with H_3_PO_4_) and acetonitrile (85:15 *v*/*v*) at 35 °C. The flow rate was 1 mL min^−1^.

### 2.5. Method Validation

The validation procedure considers the whole analytical procedure (sample pretreatment step and HPLC analysis). The proposed method was validated using rice flour as a reference material. As the target analyses were not observed on the chromatograms during the analysis of extracts of unspiked rice flour samples, this matrix was used as reference material for method validation. Rice flour samples were fortified with AMPA and NPG at different concentration levels, and extracts were obtained as described in the experiment. Real samples were spiked in the range from 0.010 mgL^−1^ to 2 mgL^−1^ The presented HPLC—PDA system was characterized by good linearity for concentration range up to 2 mg L^−1^ and the correlation coefficient above 0.998. Each spiked sample was evaluated in six replicates to assess the repeatability of the developed analytical method, and the results are shown as an average. The corresponding limits of detection and quantification (LOD and LOQ) were calculated as three and ten times the standard deviation of the noise signal, respectively.

## 3. Results and Discussion

### 3.1. Sample Pretreatment Step

The character of the real tested samples (walnuts, soybeans, buckwheat, barley groats, lentils and rice flour) forced the use of solvent extraction step before carrying out the appropriate SLM and HPLC-PDA experiments. It was required to isolate NPG and AMPA from the solid matrix. For this purpose, the ultrasound-assisted extraction technique was applied. During the optimization of the UAE process, the influence of distilled water and other solvents (mixture of methanol: water (50:50 *v*/*v*) and aqueous acetic acid solutions at a concentration of 0.1%, 0.5% and 1% was investigated. According to the polar nature of the analyzed compounds, the highest efficiency of the isolation step was observed when distilled water was used for the UAE extraction procedure. The protein precipitation stage was required because of the presence of UAE extracts, interfering substances (primarily proteins), which decreased the efficiency of the enrichment process through SLM. At this point, it should be indicated that the direct HPLC analysis of the UAE extracts was not possible due to the presence in the samples of macromolecular interfering substances (proteins).

**Figure 1:**
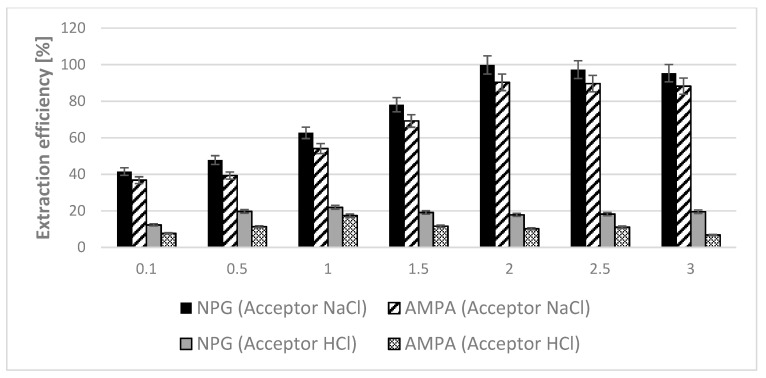
Influence of the type and concentrations of acceptor phase on the SLM extraction efficiency of tested analytes from aqueous samples. (SLM conditions—donor phase: an aqueous solution of NPG and AMPA mixture at a concentration of 0.6 mg L^−1^ and pH 11 (sample volume 100 mL); acceptor phase: NaCl or HCl solutions; membrane phase: 20% Aliquat 336 in DHE; the flow rate of the donor and acceptor phase 0.2 mL min^−1^; extraction time—24 h).

The initial parameters of membrane extraction were based on our previous work where glyphosate and AMPA were determined in the fruit juices [24]. For this case, the 0.1 M HCl was used as the acceptor phase. Thus, the same acceptor composition was chosen to enrich our UAE extracts (Figure 1). Unfortunately, it turned out that this type of acceptor phase was inappropriate for obtaining desirable enrichment of the studied analytes. The maximum extraction efficiency, for glyphosate and AMPA, with HCl as acceptor phase, was less than 20%. Only the replacement of the acceptor phase from hydrochloric acid into sodium chloride resulted in obtaining a significant increase in the extraction efficiency of the tested compounds. As can be seen, the obtained results clearly showed that the use of NaCl as the acceptor phase allowed to obtain a better enrichment effect of the tested analytes (in some cases even five times) compared to the experiments in which hydrochloric acid was used as the acceptor phase. Using sodium chloride at a concentration ≥ 2 mol L^−1^ made it possible to extract NPG with 100% extraction efficiency (E) and over 90% for AMPA. It seems that analytes extraction into the organic phase must be favoured for AMPA due to its lower polarity (AMPA logP = −2.16, NPG log P = −2.8). However, probably not only the polarity of the studied analytes affected their enrichment. In the proposed method, the transport mechanism through the liquid membrane with Aliquat 336 is counter-ion carrier-mediated transport. If no driving force is present in the system (very low chloride anion concentration), AMPA and NPG are only transported until equilibrium of the analyte concentration between donor and acceptor is established. Higher NaCl concentration increases the driving force, and in turn, higher extraction is achieved. However, a further increase in the salt present in the acceptor phase does not cause a rise in the extraction efficiency. Such a phenomenon can result from the changes in acceptor phase composition during the extraction process and can be a reason for incomplete trapping that often occurs in the liquid membrane extraction using SLM. It may suggest that for AMPA, the incomplete trapping effect is higher than in the case of glyphosate, which is in agreement with dependencies observed earlier [26,27]. Furthermore, in the case of NPG, both acidic groups (carboxylic and phosphonate) can participate in forming ion-pair with molecules of cationic carrier presented in the membrane phase. AMPA has only one acidic group in the structure. Twice as many possibilities may explain the greater efficiency of glyphosate extraction.

**Figure 2:**
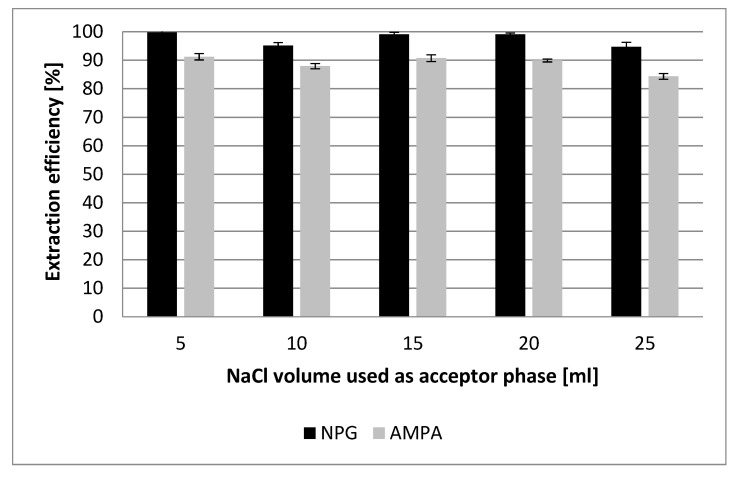
Effect of donor phase volume on extraction efficiency of the studied analytes. (SLM conditions—donor phase: an aqueous solution of NPG and AMPA mixture at a concentration of 0.6 mg L^−1^ and pH 11 (sample volume 100 mL); acceptor phase: 2 M NaCl; membrane phase: 20% Aliquat 336 in DHE; the flow rate of the donor and acceptor phase 0.2 mL min^−1^; extraction time: 24 h).

During the SLM extraction, it was necessary to use the so-called continuous flow of both the donor and acceptor phases to ensure adequate contact time of the studied analytes with the surface of the liquid membrane and carrier (Aliquat336). Furthermore, as it was mentioned earlier, increasing NaCl concentration increases the driving force. However, further change in the salt concentration in the acceptor phase does not cause a rise in the extraction efficiency. This phenomenon can be attributed to the fact that during extraction, the concentration of glyphosate and AMPA reaches the limit of solubility, and more studied analytes could not be transported. Therefore, the experiments with different volumes of used acceptor phase were performed. The obtained results indicated that reduction of the acceptor phase volume did not significantly affect the efficiency of the SLM process. In this case, the V_d_/V_a_ ratio equal to 20:1 made it possible to obtain practically 100% extraction efficiency for NPG and 90% for AMPA (Figure 2). Furthermore, as no changes were observed, the solubility limit had no effect on analyte transport across the hydrophobic liquid membrane.

Based on the conducted experiments and previous studies, the final SLM extraction parameters used in the developed extraction procedure are summarized in Table 2.

**Figure 3:**
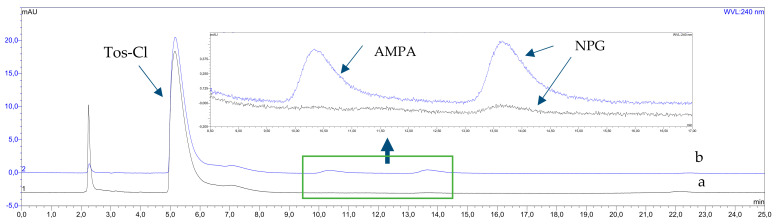
The HPLC chromatograms of the standard aqueous solution of AMPA and NPG before (**a**) and after UAE—SLM extract (**b**).

To assess the degree of recovery of the developed procedure for isolation and enrichment of NPG and AMPA, in the next stage, an analysis of the aqueous solution of the tested standard substances with a concentration of 0.6 mg L^−1^ was carried out. The HPLC-PDA analysis of the obtained UAE-SLM extracts, after derivatization with Tos-Cl, are presented in Figure 3b. As it can be seen, without an extraction procedure, only glyphosate could be determined (Figure 3a). The obtained results confirmed the effectiveness of the developed analytical method. On the other hand, using the SLM enrichment process significantly increased the extraction efficiency of both analytes. The obtained recoveries values (both for NPG and AMPA) for samples of aqueous solutions were over 97% ± 3%. The repeatability and precision of the developed results, determined based on the RSD parameter, was high and amounted to less than 3%.

### 3.2. Method Validation

The method validation was performed using the real sample for accounting he matrix effect. The selected reference material was rice flour because it was devoid of pesticides or the concentration of phosphonates was <LOD of the analytical method used. The optimized analytical procedure of UAE—SLM—HPLC—PDA was linear in the concentration range from 0.01 mg L^−1^ to 2 mg L^−1^ with linear regression coefficients (R^2^) above 0.999. The determined values of the limits of detection (LODs) and quantification (LOQs) for NPG and AMPA were respectively: 0.002 µg g^−1^ and 0.006 µg g^−1^ as well as 0.210 µg g^−1^ and 0.630 µg g^−1^. As can be seen, the LODs and LOQs values were significantly higher for glyphosate than for AMPA. Unfortunately, the polar nature of AMPA significantly reduced the efficiency of its separation and enrichment using the developed UAE-SLM extraction procedure. Results obtained in the validation experiments are presented in Table 3.

The obtained LODs values of the developed method are comparable to those results described in the literature (Table 4) in which detection with a mass spectrometer is used.

### 3.3. Food Sample Analysis

Finally, attempts were made to analyze real samples—food samples. For this case, six different types of solid food products, potentially containing residues of the tested phosphonates, were selected. The analyzed food consisted of walnut, soybean, barley, buckwheat groats, lentils and rice flour. Extraction of food samples showed different recovery efficiency of the tested analytes, depending on the composition of their matrix (Table 5).

The obtained values of recoveries ranged from 32% to 69% for NPG and from 29% to 56% for AMPA. For samples such as walnut and soybeans, which contained significant amounts of fat, the recoveries obtained were the lowest (less than 50%). Moreover, in all experiments, the obtained recovery of glyphosate was higher compared to those obtained for AMPA, which was directly related to their different polarity.

**Figure 4:**
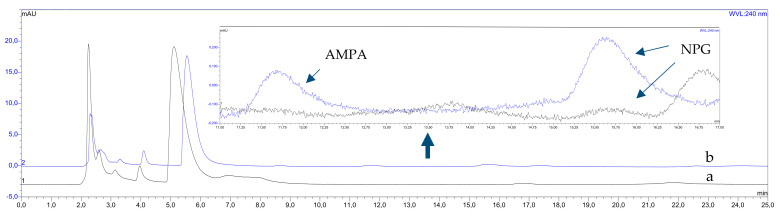
Chromatograms obtained from the analysis of UAE—SLM extracts of rice flour samples: (**a**)—blank sample and (**b**)—fortified extract (SLM conditions—donor: 100 mL of extract at pH = 11; acceptor: 5 mL 2 M NaCl; membrane phase: 20% Aliquat 336 in DHE; flow rate of donor and acceptor phases: 0.2 mL/min; extraction time: 24 h).

**Figure 5:**
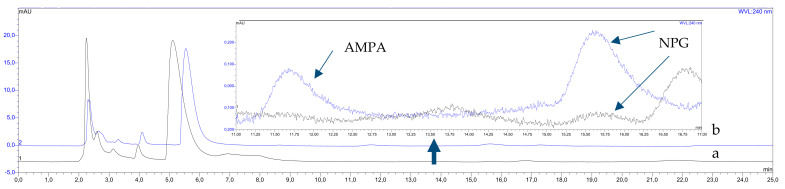
Chromatograms obtained from the analysis of UAE—SLM extracts of barley groats samples: (**a**)—blank sample and (**b**)—fortified extract (SLM conditions—donor: 100 mL of extract at pH = 11, acceptor: 5 mL 2 M NaCl, membrane phase: 20% Aliquat 336 in DHE, flow rate of donor and acceptor phases: 0.2 mL/min, extraction time: 24 h).

Figure 4 and Figure 5 show as an example the chromatograms obtained from the analysis of selected food samples of rice flour (Figure 4) and barley (Figure 5), without the addition of a standard (the so-called reference material, blank sample (Figure 4a and Figure 5a), and fortified samples (Figure 4b and Figure 5b), respectively. In this case, it can be seen that the use of the extraction procedure made it possible to isolate and enrich the tested compounds with high efficiency. In addition, an exemplary analysis of barley groats samples (spiked and unspiked samples) showed the presence of glyphosate in the tested food material. As a result, 4 out of 6 analyzed matrices contained NPG, at the concentration of which could be quantified. The lentils (11.0 ± 0.6 µg/kg) had the highest NPG content, followed by walnut (7.8 ± 0.2 µg/kg), barley and soybeans (6.5 ± 0, respectively, 2 µg/kg and 4.7 ± 0.1 µg/kg). In the case of buckwheat and rice flour samples, no glyphosate was found at the concentration level of the analytical procedure used. Moreover, no AMPA residues were observed in the analyzed food samples in any case.

According to the analysis of Glyphosate residues in foods from the Canadian Retail Markets between 2015 and 2017 [18], the maximum observed concentration of glyphosate was 0.45 ppm, which is almost 10 times below the MRL of 3 ppm. For lentils the level of the determined NPG was between 0.021–2.6 ppm. In barley it was 0.098 ppm, whereas dried soybeans contain 0.0069–0.024 ppm of NPG. For buckwheat (grain) it was 0.006–0.33 ppm and rice (flour) 0.007–0.034 ppm. As it can be seen the developed method make possible the determination of NPG and AMPA at requiered level.

## 4. Conclusions

Glyphosate is among the universally used pesticides in the world. Glyphosate is currently still authorized for use in both Germany and the European Union (EU) as a plant protector substance for the combating of weed. There is a tendency towards more frequent determinations of glyphosate in certain food groups, including lentils, linseeds, buckwheat and millet in the cereals and cereal products category, as well as in legumes. According to the U.S. Food and Drug Administration (FDA) Pesticide Residue Monitoring Report from 2018 glyphosate was determined in over 60% of corn samples, and 67% of soybean samples. A high frequency of detection can be seen here in buckwheat (15%). From a total of 473 samples from the category legumes, oil seeds, nuts, and soy products, 40 samples (9%) were positive for glyphosate in amounts > 0.02 mg/kg. Exceedances of the MRLs occurred in 15 samples (about 3%). A high frequency of NPG findings was observed in peanuts (13%), chick peas (11%), linseeds (21%) and lentils (15%), walnuts (6%). Only 27 samples contained glyphosate amounts exceeding the respective, legal, EU-wide harmonized MRLs, which accounts for a violation rate of just 0.16%.

In the case of the developed UAE—SLM—HPLC—PDA method, the costs of the process are significantly reduced, and the need for highly selective detection methods such as MS detection are eliminated. Furthermore, there are not many analytical methods available to determine glyphosate in such samples, as was presented in this work. The tested matrices are characterized by a very complex matrix composition, which is a significant analytical challenge.

## Figures and Tables

**Table 1:** Characteristic of the analyzed food samples according to information obtained from product labels.

Type of Sample	Nutritional Factor 100 g of Product [g]				
	Protein	Carbohydrates	Fats	Cellulose	Sodium
Walnut	5.6	16.6	16.0	<0.5	0.030
Soybean	34.2	19.6	17.0	15.7	0.001
Buckwheat	14.1	72.4	1.4	-	-
Barley	10.6	66.0	2.1	9.1	0.001
Lentil	24.4	45.7	1.9	8.9	0.012
Rice flour	7.9	79.0	0.9	0.7	0.001

**Table 2:** The selected parameters of SLM extraction of the tested analytes.

Parameter	SLM
Membrane phase	20% Aliquat 336 in DHE
Donor phase	Sample volume 100 mL and pH 11
Acceptor phase	2 mol L^−1^ NaCl, volume 5 mL
Donor phase flow rate	0.2 mL/min.
Acceptor phase flow rate	0.2 mL/min.
Extraction time	24 h

**Table 3:** Results obtained in the validation experiments.

Matrix	R^2^	LODs and LOQs	RSD [%] (*n* = 6)	HPLC—PDA Method Precision	RSD [%] Beetwen Days
RICE FLOUR	>0.999	LODs: 0.002 µg g^−1^ for NPG 0.210 µg g^−1^ for AMPA LOQs: 0.006 µg g^−1^ for NPG 0.630 µg g^−1^ for AMPA	≤6%	<5% for peak area <0.2% for retention time	0.5–4%

R^2^—the linear regression coefficient.

**Table 4:** Analytical performance of the developed analytical procedure compared to the other methods used for the determination of NPG and AMPA in food samples.

Sample Matrix	Sample Pretreatment Step	Analysis	LOD/LOQ	Ref.
carrot	LLE	GC-FPD, derivatization with N-isoPOC	LOD NPG = 12 pg LOD AMPA = 8 pg	[28]
guava fruit	SPE sorbent with Fe_2_O_3_—Al_2_O_3_ nanoparticles, MIP)	CE—ECL	LOD NPG = 0.01 ug g^−1^	[29]
soybean	UAE solvent extraction	CE—ECL	LOD NPG = 0.6 ug g^−1^	[30]
soybean	LLE	LC/ESI—MS/MS	LOD NPG = 0.09 ug g^−1^ LOD AMPA = 0.1 ug g^−1^	[31]
honey	SPE with Oasis HLB extraction cartridge	LC-MS/MS	LOD NPG = 0.001 ug g^−1^ LOD AMPA = 0.001 ug g^−1^	[32]
amaranth, barley, oat, and quinoa	QuEChERS extraction method	Multicommutated Flow System Based on Its Quenching Effect on CdTe-Quantum Dots Fluorescence	LOD NPG = 0.5 ug mL^−1^	[33]
walnut, soybean, buckwheat, barley, lentil, rice flour	UAE-SLM ,Protein precipitation	HPLC—PDA	LOD NPG = 0.002 ug g^−1^ LOD AMPA = 0.210 ug g^−1^	***

AMPA—aminomethyl phosphonic acid; CE-ECL—capillary electrophoresis with enhanced chemiluminescence detection; ESI- electrospray ionization; GC-FPD—gas chromatography with Flame Photometric Detector; HPLC—high performance liquid chromatography; LLE—liquid-liquid extraction; LOD—limit of detection; LOQ—limit of quantification; MIP—molecularly imprinted polymer; MS/MS—tandem mass spectrometry; N-isoPOC—*N*-isopropoxycarbonyl; NPG—*N*-(phosphonomethyl) glycine; Oasis-HLB —the SPE cartridges containing the *Oasis HLB* sorbent; PDA—photodiode array detector; SPE—solid-phase extraction; SLM—supported liquid membranes; QuEChERS—the acronym for a highly beneficial analytical approach that vastly simplifies the analysis of multiple pesticide residues in complex sample matrices; UAE—ultrasonic-assisted solvent extraction; *** This work.

**Table 5:** Determined values of NPG and AMPA concentration in the tested samples.

Type of Sample	NPG and AMPA Analysis of Tested Food Samples, *n* = 6			
	Recovery NPG [%]	Recovery AMPA [%]	Determined Content of NPG [µg/kg]	Determined Content of AMPA [µg/kg]
Walnut	32.1 ± 0.7	28.8 ± 1.0	7.8 ± 0.2	<LOD
Soybean	48.7 ± 1.3	35.1 ± 6.0	4.7 ± 0.1	<LOD
Buckwheat	69.0 ± 0.5	49.0 ± 2.1	< LOD	<LOD
Barley	46.8 ± 1.3	37.4 ± 1.5	5.6 ± 0.2	<LOD
Lentil	67.1 ± 3.6	36.5 ± 5.8	11.0 ± 0.6	<LOD
Rice flour	61.9 ± 0.8	55.9 ± 4.9	<LOD	<LOD
